# Supplementary material for: HIVprotI: an integrated web based platform for prediction and design of HIV proteins inhibitors
Source: J Cheminform. 2018 Mar 9;10:12. doi: 10.1186/s13321-018-0266-y (PMC5845081; doi:10.1186/s13321-018-0266-y)
Supplement: Supplementary file 2 — Additional file 2. Source code of HIVProtI web server. [file 13321_2018_266_MOESM2_ESM.zip › HIVprotI_Source-code/hivprotI/weka-3-6-6/doc/weka/attributeSelection/CheckAttributeSelection.html]

CheckAttributeSelection


---


|  |  |  |
| --- | --- | --- |
| **Overview** \| **Package** \| **Class** \| **Tree** \| **Deprecated** \| **Index** \| **Help** \| **Weka's home** | |  |
| **PREV CLASS**   **NEXT CLASS** | **FRAMES**    **NO FRAMES**     **All Classes** |  |
| SUMMARY: NESTED \| FIELD \| CONSTR \| METHOD | DETAIL: FIELD \| CONSTR \| METHOD |  |


---


## weka.attributeSelection Class CheckAttributeSelection

```
java.lang.Object
  weka.core.Check
      weka.core.CheckScheme
          weka.attributeSelection.CheckAttributeSelection
```

**All Implemented Interfaces:**: OptionHandler, RevisionHandler

---

``` public class CheckAttributeSelection extends CheckScheme ```

Class for examining the capabilities and finding problems with
attribute selection schemes. If you implement an attribute selection using
the WEKA.libraries, you should run the checks on it to ensure robustness
and correct operation. Passing all the tests of this object does not mean
bugs in the attribute selection don't exist, but this will help find some
common ones.

Typical usage:

`java weka.attributeSelection.CheckAttributeSelection -W ASscheme_name
-- ASscheme_options` 

CheckAttributeSelection reports on the following:

- Scheme abilities
  - Possible command line options to the scheme
  - Whether the scheme can predict nominal, numeric, string,
    date or relational class attributes.
  - Whether the scheme can handle numeric predictor attributes
  - Whether the scheme can handle nominal predictor attributes
  - Whether the scheme can handle string predictor attributes
  - Whether the scheme can handle date predictor attributes
  - Whether the scheme can handle relational predictor attributes
  - Whether the scheme can handle multi-instance data
  - Whether the scheme can handle missing predictor values
  - Whether the scheme can handle missing class values
  - Whether a nominal scheme only handles 2 class problems
  - Whether the scheme can handle instance weights
- Correct functioning
  - Correct initialisation during search (i.e. no result
    changes when search is performed repeatedly)
  - Whether the scheme alters the data pased to it
    (number of instances, instance order, instance weights, etc)
- Degenerate cases
  - building scheme with zero instances
  - all but one predictor attribute values missing
  - all predictor attribute values missing
  - all but one class values missing
  - all class values missing

Running CheckAttributeSelection with the debug option set will output the
training dataset for any failed tests.

The `weka.attributeSelection.AbstractAttributeSelectionTest`
uses this class to test all the schemes. Any changes here, have to be
checked in that abstract test class, too.


Valid options are:

```
 -D
  Turn on debugging output.
```

```
 -S
  Silent mode - prints nothing to stdout.
```

```
 -N <num>
  The number of instances in the datasets (default 20).
```

```
 -nominal <num>
  The number of nominal attributes (default 2).
```

```
 -nominal-values <num>
  The number of values for nominal attributes (default 1).
```

```
 -numeric <num>
  The number of numeric attributes (default 1).
```

```
 -string <num>
  The number of string attributes (default 1).
```

```
 -date <num>
  The number of date attributes (default 1).
```

```
 -relational <num>
  The number of relational attributes (default 1).
```

```
 -num-instances-relational <num>
  The number of instances in relational/bag attributes (default 10).
```

```
 -words <comma-separated-list>
  The words to use in string attributes.
```

```
 -word-separators <chars>
  The word separators to use in string attributes.
```

```
 -eval name [options]
  Full name and options of the evaluator analyzed.
  eg: weka.attributeSelection.CfsSubsetEval
```

```
 -search name [options]
  Full name and options of the search method analyzed.
  eg: weka.attributeSelection.Ranker
```

```
 -test <eval|search>
  The scheme to test, either the evaluator or the search method.
  (Default: eval)
```

```
 Options specific to evaluator weka.attributeSelection.CfsSubsetEval:
```

```
 -M
  Treat missing values as a seperate value.
```

```
 -L
  Don't include locally predictive attributes.
```

```
 Options specific to search method weka.attributeSelection.Ranker:
```

```
 -P <start set>
  Specify a starting set of attributes.
  Eg. 1,3,5-7.
  Any starting attributes specified are
  ignored during the ranking.
```

```
 -T <threshold>
  Specify a theshold by which attributes
  may be discarded from the ranking.
```

```
 -N <num to select>
  Specify number of attributes to select
```

**Version:**
:   $Revision: 4783 $

**Author:**
:   Len Trigg (trigg@cs.waikato.ac.nz), FracPete (fracpete at waikato dot ac dot nz)

**See Also:**: `TestInstances`

---

**Nested Class Summary**

**Nested classes/interfaces inherited from class weka.core.CheckScheme**

`CheckScheme.PostProcessor`


**Constructor Summary**

`CheckAttributeSelection()`


| **Method Summary** | |
| --- | --- |
| `void` | `doTests()`             Begin the tests, reporting results to System.out |
| `ASEvaluation` | `getEvaluator()`             Get the current evaluator |
| `java.lang.String[]` | `getOptions()`             Gets the current settings of the CheckAttributeSelection. |
| `java.lang.String` | `getRevision()`             Returns the revision string. |
| `ASSearch` | `getSearch()`             Get the current search method |
| `boolean` | `getTestEvaluator()`             Gets whether the evaluator is being tested or the search method. |
| `java.util.Enumeration` | `listOptions()`             Returns an enumeration describing the available options. |
| `static void` | `main(java.lang.String[] args)`             Test method for this class |
| `void` | `setEvaluator(ASEvaluation value)`             Set the evaluator to test. |
| `void` | `setOptions(java.lang.String[] options)`             Parses a given list of options. |
| `void` | `setSearch(ASSearch value)`             Set the search method to test. |
| `void` | `setTestEvaluator(boolean value)`             Sets whether the evaluator or the search method is being tested. |

**Methods inherited from class weka.core.CheckScheme**

`attributeTypeToString, getNumDate, getNumInstances, getNumInstancesRelational, getNumNominal, getNumNumeric, getNumRelational, getNumString, getPostProcessor, getWords, getWordSeparators, hasClasspathProblems, setNumDate, setNumInstances, setNumInstancesRelational, setNumNominal, setNumNumeric, setNumRelational, setNumString, setPostProcessor, setWords, setWordSeparators`

**Methods inherited from class weka.core.Check**

`getDebug, getSilent, setDebug, setSilent`

**Methods inherited from class java.lang.Object**

`equals, getClass, hashCode, notify, notifyAll, toString, wait, wait, wait`

**Constructor Detail**

### CheckAttributeSelection

```
public CheckAttributeSelection()
```


**Method Detail**

### listOptions

```
public java.util.Enumeration listOptions()
```

:   Returns an enumeration describing the available options.

    :   **Specified by:**: `listOptions` in interface `OptionHandler` **Overrides:**: `listOptions` in class `CheckScheme`
    :   **Returns:**: an enumeration of all the available options.

---


### setOptions

```
public void setOptions(java.lang.String[] options)
                throws java.lang.Exception
```

:   Parses a given list of options.


    Valid options are:

    ```
     -D
      Turn on debugging output.
    ```

    ```
     -S
      Silent mode - prints nothing to stdout.
    ```

    ```
     -N <num>
      The number of instances in the datasets (default 20).
    ```

    ```
     -nominal <num>
      The number of nominal attributes (default 2).
    ```

    ```
     -nominal-values <num>
      The number of values for nominal attributes (default 1).
    ```

    ```
     -numeric <num>
      The number of numeric attributes (default 1).
    ```

    ```
     -string <num>
      The number of string attributes (default 1).
    ```

    ```
     -date <num>
      The number of date attributes (default 1).
    ```

    ```
     -relational <num>
      The number of relational attributes (default 1).
    ```

    ```
     -num-instances-relational <num>
      The number of instances in relational/bag attributes (default 10).
    ```

    ```
     -words <comma-separated-list>
      The words to use in string attributes.
    ```

    ```
     -word-separators <chars>
      The word separators to use in string attributes.
    ```

    ```
     -eval name [options]
      Full name and options of the evaluator analyzed.
      eg: weka.attributeSelection.CfsSubsetEval
    ```

    ```
     -search name [options]
      Full name and options of the search method analyzed.
      eg: weka.attributeSelection.Ranker
    ```

    ```
     -test <eval|search>
      The scheme to test, either the evaluator or the search method.
      (Default: eval)
    ```

    ```
     Options specific to evaluator weka.attributeSelection.CfsSubsetEval:
    ```

    ```
     -M
      Treat missing values as a seperate value.
    ```

    ```
     -L
      Don't include locally predictive attributes.
    ```

    ```
     Options specific to search method weka.attributeSelection.Ranker:
    ```

    ```
     -P <start set>
      Specify a starting set of attributes.
      Eg. 1,3,5-7.
      Any starting attributes specified are
      ignored during the ranking.
    ```

    ```
     -T <threshold>
      Specify a theshold by which attributes
      may be discarded from the ranking.
    ```

    ```
     -N <num to select>
      Specify number of attributes to select
    ```

    :   **Specified by:**: `setOptions` in interface `OptionHandler` **Overrides:**: `setOptions` in class `CheckScheme`
    :   **Parameters:**: `options` - the list of options as an array of strings **Throws:**: `java.lang.Exception` - if an option is not supported

---


### getOptions

```
public java.lang.String[] getOptions()
```

:   Gets the current settings of the CheckAttributeSelection.

    :   **Specified by:**: `getOptions` in interface `OptionHandler` **Overrides:**: `getOptions` in class `CheckScheme`
    :   **Returns:**: an array of strings suitable for passing to setOptions

---


### doTests

```
public void doTests()
```

:   Begin the tests, reporting results to System.out

    :   **Specified by:**: `doTests` in class `CheckScheme`

---


### setEvaluator

```
public void setEvaluator(ASEvaluation value)
```

:   Set the evaluator to test.

    :   **Parameters:**: `value` - the evaluator to use.

---


### getEvaluator

```
public ASEvaluation getEvaluator()
```

:   Get the current evaluator

    :   **Returns:**: the current evaluator

---


### setSearch

```
public void setSearch(ASSearch value)
```

:   Set the search method to test.

    :   **Parameters:**: `value` - the search method to use.

---


### getSearch

```
public ASSearch getSearch()
```

:   Get the current search method

    :   **Returns:**: the current search method

---


### setTestEvaluator

```
public void setTestEvaluator(boolean value)
```

:   Sets whether the evaluator or the search method is being tested.

    :   **Parameters:**: `value` - if true then the evaluator will be tested

---


### getTestEvaluator

```
public boolean getTestEvaluator()
```

:   Gets whether the evaluator is being tested or the search method.

    :   **Returns:**: true if the evaluator is being tested

---


### getRevision

```
public java.lang.String getRevision()
```

:   Returns the revision string.

    :   **Returns:**: the revision

---


### main

```
public static void main(java.lang.String[] args)
```

:   Test method for this class

    :   **Parameters:**: `args` - the commandline parameters


---


|  |  |  |
| --- | --- | --- |
| **Overview** \| **Package** \| **Class** \| **Tree** \| **Deprecated** \| **Index** \| **Help** \| **Weka's home** | |  |
| **PREV CLASS**   **NEXT CLASS** | **FRAMES**    **NO FRAMES**     **All Classes** |  |
| SUMMARY: NESTED \| FIELD \| CONSTR \| METHOD | DETAIL: FIELD \| CONSTR \| METHOD |  |


---
